# The Role of the Plant–Soil Relationship in Agricultural Production—With Particular Regard to PGPB Application and Phytoremediation

**DOI:** 10.3390/microorganisms11061616

**Published:** 2023-06-19

**Authors:** Szilvia Kisvarga, Dóra Hamar-Farkas, Máté Ördögh, Katalin Horotán, András Neményi, Dezső Kovács, László Orlóci

**Affiliations:** 1 Ornamental Plant and Green System Management Research Group, Institute of Landscape Architecture, Urban Planning and Garden Art, Hungarian University of Agriculture and Life Sciences (MATE), 1223 Budapest, Hungary; farkas.dora@uni-mate.hu (D.H.-F.); nemenyi.andras.bela@uni-mate.hu (A.N.); orloci.laszlo@uni-mate.hu (L.O.); 2 Department of Floriculture and Dendrology, Institute of Landscape Architecture, Urban Planning and Garden Art, Hungarian University of Agriculture and Life Sciences (MATE), 1118 Budapest, Hungary; ordogh.mate@uni-mate.hu (M.Ö.); kovacsdezso.zsztgy@gmail.com (D.K.); 3 Zoological Department, Institute of Biology, Eszterházy Károly Catholic University, 3300 Eger, Hungary; horotan.katalin@uni-eszterhazy.hu

**Keywords:** PGPB, plant growth-promoting bacteria, plant, stress, microbes, rhizosphere, soil rehabilitation, soil microbial communities, omics, consortism

## Abstract

Plant growth-promoting bacteria (PGPB) and other living organisms can help with the challenges of modern agriculture. PGPB offer ever-expanding possibilities for science and commerce, and the scientific results have been very advanced in recent years. In our current work, we collected the scientific results of recent years and the opinions of experts on the subject. Opinions and results on soil–plant relations, as well as the importance of PGPB and the latest related experiences, are important topics of our review work, which highlights the scientific results of the last 3–4 years. Overall, it can be concluded from all these observations that the bacteria that promote plant development are becoming more and more important in agriculture almost all over the world, thus, promoting more sustainable and environmentally conscious agricultural production and avoiding the use of artificial fertilizers and chemicals. Since many mechanisms of action, namely biochemical and operational processes, are still under investigation, a new emerging scientific direction is expected in the coming years with regard to PGPB, microbial, and other plant growth-stimulating substances, in which omics and microbial modulation also play a leading role.

## 1. Introduction

**Figure 1:**
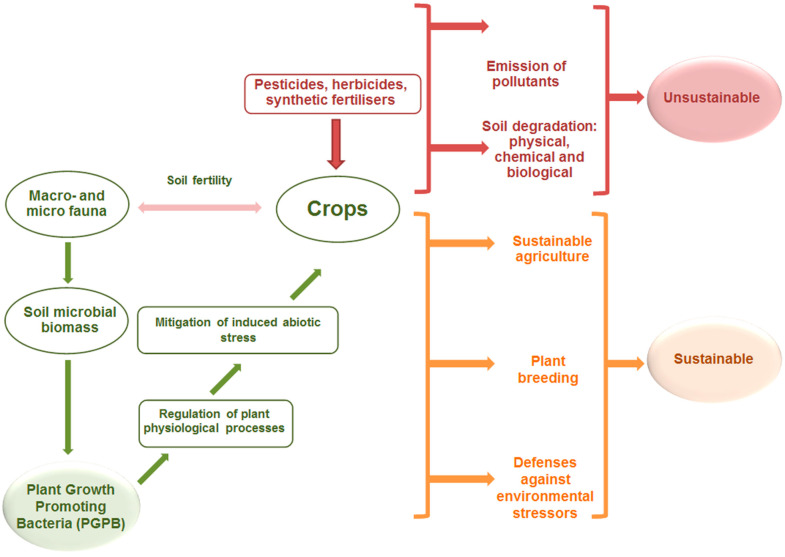
Place of PGPB groups in biomes and benefits from their use (Horotán, 2023).

Soil microbiomes can be considered the beginning of the next generation green revolution, as plant growth-promoting bacteria (PGPB) play a prominent role in planning sustainable agriculture with microbiomes [1,2,3]. PGPB may be valid sources of secondary metabolites that are useful in sustainable agriculture [4]. Among others, these bacteria groups include strains from genera *Paenibacillus*, *Azospirillum*, *Rhizobium*, *Bacillus*, *Azotobacter*, *Klebsiella*, *Pseudomonas*, and *Serratia* [5]. In addition to the nutrient content, as well as their ability suppress the infection of pathogens [4,6,7], they also positively influence the production of vitamins, phytohormones, and growth regulators [5], and modify the concentration of certain phytohormones involved in the plant–PGPB relationship. A significant increase in the effective use of PGPB and a concomitant reduction in the use of potentially harmful chemicals requires a better understanding of how these beneficial bacteria function and interact with plants [8]. Several options are available for in situ labeling of microorganisms. The purpose of this is to research their properties (e.g., in situ hybridization, stable isotope probing, substrate analog testing, fluorescence) [9]. There is increasing interest in approaching PGPB through omics because some of the limitations of cultivation-based studies have been overcome by being more closely linked with molecular techniques [10]. In recent years, with the continuous development of omics techniques (genomics, transcriptomics, proteomics, ionomics, and metabolomics), we are moving closer and closer to understanding the interactions between plants and microorganisms [11]. Many results have been achieved on the subject in recent years, but Norstedt and Jones [12] also described that a holistic approach to microbe–soil interaction is currently lacking. Over the years, many PGPB genera have been isolated and studied. Several new bacterial genera may also be suitable for use as PGPB [13] (Figure 1).

However, only a few of them are commercially available, mainly due to the unsuccessful survival of bacteria after application in formulations and agroecosystems [4]. The metagenomics results may lead to new PGPB species being researched, as Thrash [14] isolated TM7 and SAR1 strains using fluorescence methods and antibodies targeting cell surface proteins. The Raman-activated microbial cell sorting method is capable of sorting cells containing a labeled substrate sufficient to generate a Raman peak. With this, the viability of the cells is preserved, so it is also suitable for isolating cells that play a role in metabolic processes [15]. In addition to the Raman method, bio-orthogonal non-canonical amino acid labeling (BONCAT) is a valuable new method for identifying organisms based on activity [16].

It is generally accepted that the effectiveness of PGPB depends on their ability to establish in an environment and compete with native plant microorganisms under agricultural conditions [17]. Strains introduced to manipulate microbiomes are usually eliminated in the soil, but more and more publications are also showing that the use of PGPB as an inoculant is effective in promoting plant growth [2]. The identification of molecules and their genes involved in the mediation of plant–microbe interactions opens the way to improve plant cultivation and to manipulate plant–microbe interactions mediated by metabolites [18]. To monitor this, reporter genes, immunological reactions, and nucleic acid research methods are used for the PGPB markers found in seeds, plants, or soil [17]. The primary cause of antibiotic resistance in PGPB is the presence of antibiotic resistance genes and intrinsic resistance from efflux pumps [6].

**Figure 2:**
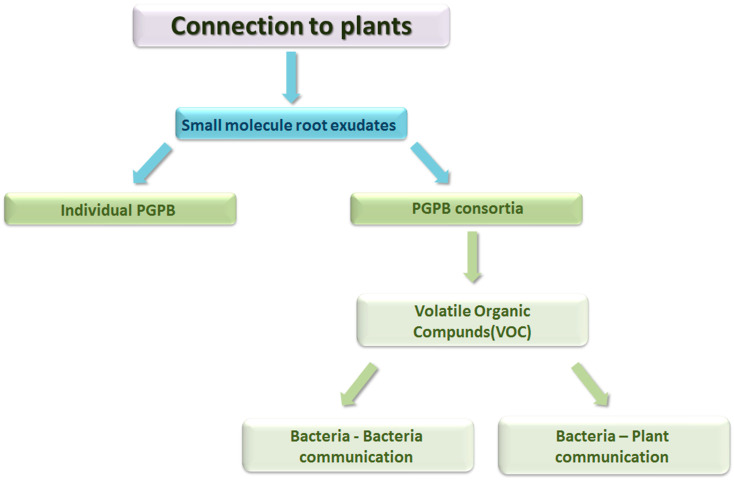
Plant relationship of PGPB groups (Horotán, 2023).

In order to map strains that are effective for plants, it is necessary to study certain strains of the PGPB in relation to the given soil and climatic conditions [19], because strain specificity is a tool for understanding the characteristic potential of microbes [20]. In soil, PGPB usually work together in consortia. These groups promote plant development. Within the consortium, the bacteria have different tasks. The bacterial specificity is determined by the root exudate of the plant, namely its composition, quantity, and concentration [8]. Currently, two types of consortia are known: simple and complex consortia. The difference between them lies in the fermentation strategy, during which the strains are propagated alone or in combination with other strains and other species [21] (Figure 2).

Volatile organic compounds (VOCs) are also signal compounds in consortia, which have a role in both bacteria–bacteria and plant–bacteria interactions [22]. Optimizing the bacterial interaction in a consortium may depend on several factors. Among them is a sufficiently deep knowledge and identification strategy of the consortium, as well as the possibility of its creation. In addition, the properties of the soil and the size of the populations of the species participating in the consortium are also important [23].

Certain phytohormones, such as ethylene, salicylic acid, and indole-3-acetic acid, regulate plant–microbe interactions. For this reason, the knowledge and application of phytohormone-degrading bacteria is crucial for the development of novel solutions aimed at the growth and protection of plants. The possibility of modifying the concentration of the phytohormone can be a guide in the colonization of bacteria and, thus, in the promotion of plant development [24]. DBD plasma can accelerate the activity of CB-R05 bacteria by enhancing related gene expression. Colonization in the root system will be more effective as a result. As a result of the process, the phytohormone concentration level also improves, which leads to increased plant development and vitality [25].

### 1.1. Functional Identification Technologies and Results

This scientific discipline began to develop rapidly. In the last few years, high-throughput transcriptional sequencing has revealed the identification of the function of microbes [26]. Alternative splicing as a post-transcriptional process has become a prominent process against abiotic stress tolerance in plant breeding. Alternative splicing can create multiple transcripts from a single gene [27], and agriculturally effective PGPB strains have also been isolated from various plant (e.g., rhizosphere) compartments [17]. Chen et al. [2] found that PGPB also affects DNA methylation in plant roots, thus, offering new research opportunities on the topic of plant–microbiome interactions.

Molecular methods suitable for direct DNA analysis are often used to investigate the microbiome diversity of complex soil communities. This is necessary because propagation technologies are not suitable for this [28]. The applicable metagenomic studies (targeted or shotgun approach) depend on the type of environmental studies [29]. Whole genome sequencing is also suitable for characterizing PGPB [8]. The most commonly used analysis is based on the amplification of the bacterial 16S rRNA genes and the fungal ITS region. The DNA is extracted from the soil or rhizosphere, after which the bacterial and fungal species are identified from the PCR product [30]. Using this method, Li et al. [31] examined the sequence analysis of the housekeeping genes (16S rRNA and rpoB) and the nif H gene showed that the LC-T2 T strain could be a new species of the genus *Paenibacillus*.

Many multifunctional and agriculturally important microbes are found in plant internal tissues [32]. These endophytic bacteria are able to reduce stress in plants, but there are conditions for this, such as the presence and amount of volatile and bioactive compounds (ethylene, auxin plant hormones, phenols, polysaccharides, sidephores and organic acids) [33]. Amplified gene fragments can be identified and analyzed by several DNA fingerprinting techniques. Examples include terminal restriction fragment length polymorphism, temperature gradient gel electrophoresis, amplified rDNA restriction analysis, or automated ribosomal intergenic spacer analysis, if the goal is to analyze highly variable regions on the 16S and 23S DNA sections. Currently, NGS metagenomic techniques are also becoming suitable to replace DNA fingerprinting techniques [34].

Gene expression research focused on plant–PGPB relationships. In potato, the transcriptional analysis of *Burkholderia phytofirmans* PsJN examined stress-activated genes that had an impact on transcriptional regulation, reactive oxygen reduction, and homeostasis [35]. In corn and rice individuals, metabolomics was used to reveal the exo-metabolome of strains functioning as PGPB (*Pseudomonas putida* IDE-01, *Azospirillum brasilense* IDE-06 and *Bacillus megaterium* IDE-14) [36].

Accordingly, sequencing, mass spectrometry, and other metabolomics techniques can greatly facilitate the identification of high-throughput molecules for the efficient use of PGPB [37].

Arbuscular mycorrhizal fungi (AMF) can establish symbiosis with the roots of species-specific plants. These microorganisms contribute to short-term carbon sequestration in phytoremediation systems [1]. In the coexistence of fungal-absorbing hyphae and bacterial strains, transcriptomic studies can help in examining the expression levels of genes. These consortia may be suitable as biostimulators in horticulture and agriculture. However, their colonization capacity, efficiency, and flexibility must be evaluated [38]. During phenotypic, proteomic, and biochemical measurements, the results of bilateral (wheat–PGPB) and tripartite (wheat–PGPB–AMF) interactions showed that AMF and *Aspergillus brasiliensis* interaction is suitable for promoting plant growth by activating nitrogen assimilation and photosynthesis, thus, increasing amino acid and glucose levels. The PGPB–AMF interaction also proved to be proactive in infected wheat (biosynthesis of resistance-related proteins, jasmonic acid biosynthesis) [39].

### 1.2. Role of PGPB in Phytoremediation

**Figure 3:**
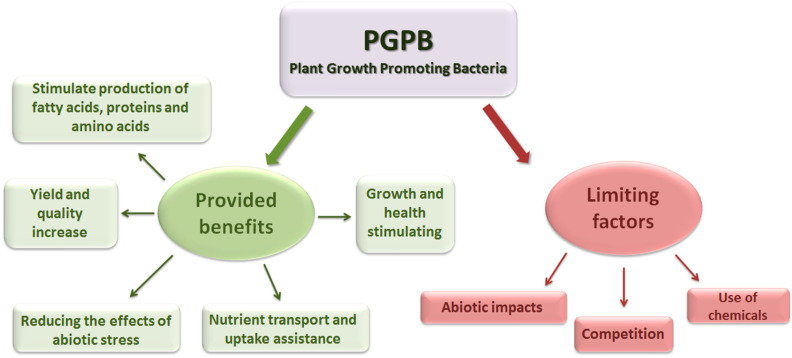
The benefits provided by PGPB groups and the most important factors inhibiting their effect (Horotán, 2023).

The phytoremediation process using only plants is time-consuming and can reduce metal uptake in the case of large amounts of pollutants [1,40]. The role of plant–microbe interactions is prominent in the adaptation of plants to metallic environments, in stimulating plant growth, so these microbe and interactions can be explored to accelerate microbial-assisted phytoremediation [1]. The effectiveness of microbial-based phytoremediation loses its effectiveness due to the high concentration of secondary toxins, lack of suitable sinks, limited access to nutrients, and the obstructed release of microbial inoculants and the lack of habitats for planktonic organisms [41]. Strains of plant microbial communities living in harsh conditions provide the research direction aimed at growing plants in extreme habitats [42] (Figure 3).

Many studies show that microbe–crop interactions play a prominent role in suboptimal adaptation, persistence, and survival, as well as soil health under multiple abiotic stressors (such as drought, salinity, UV radiation, and heavy metal pollution). Change in biochemical, physiological, and molecular processes is typical in the plants [20,43]. Promising results have been achieved in the subject in recent years. Norstedt and Jones [12] demonstrated the role of the *Serratia plymuthica* MBSA-MJ1 strain in promoting the vigor and development of several ornamental plant species (*Viola* x *wittrockiana*, *Petunia* x *hybrida*, *Impatiens walleriana*). Dobrzyński et al. [44] showed that *Bacillus pumilus* W8, *B. pumilus* LZP02, *B. pumilus* JPVS11, *B. pumilus* TUAT-1, *B. pumilus* TRS-3, and *B. pumilus* EU927414 strains also stimulate the production of fatty acids, proteins, amino acids and promoted the formation of a higher proportion of oxidative proteins in the plant organism. Certain PGPB species or strains also have an influence on the nutrient transport system of the roots. *Bacillus* spp., in the case of *Arabidopsis thaliana*, induced the expression of genes regulating nitrate and ammonium uptake and transport [45]. *Aspergillus niger* MJ1, *Pseudomonas stutzeri* DSM4166, and *P. fluorescens* CHA0-nif mutant strains had a beneficial effect on the development of lettuce and cucumber [46]. The genus *Burkholderia* can be used promisingly as an antagonizing biocontrol agent, and foe soil bioremediation and plant growth-promoting purposes [47]. Certain microorganisms can also promote the uptake of iron (they convert Fe^3+^ into Fe^2+^), and they also play a central role in metabolic functions [48]. This transformation is carried out by siderophores, proteins synthesized by bacteria, which can adsorb several metal compounds, thereby increasing their plant absorption [49]. Some microbes can produce sulfuric acid (which dissolves metals) and increase the bioavailability of sulfur (S) [50]. In combination with metal-tolerant microbiomes, *Larrea divaricata* contributes to reducing the availability and dispersion of heavy metals in contaminated soil, and improves soil structure, organic matter content, and functionality [51].

The GIC41 strain of *Lysinibacillus* significantly increased the dry weight of the shoot length on spinach plants and similarly strengthened vitality as a correlating amount of fertilizer [52]. *Enterobacter* 15S vary between maize and cucumber plants under specific phosphorus supply. Bacterial strain 15S induced cucumbers to cope with phosphorus deficiency by solubilizing an external source of insoluble phosphorus, leading to changes in root structure, mineral nutrient uptake, root efflux of phenolics and flavonoids, and a starvation-inducible increase in phosphorus levels via Pi transporter genes [53]. In the case of corn, *Bacillus mojavensis*, *B. subtilis*, and *B. pumilus* increased the yield by more than 10%, and *B. pseudomycoides* by 9.8%, compared to the control groups. The fruits of plants treated with *B. mojavensis, B. subtilis*, and *B. pumilus* strains had the highest protein and fiber content. Furthermore, high values of photosynthetic rate, transpiration rate, and stomatal conductance mostly resulted in a high yield [19]. During the examination of the native bacteria associated with the corn rhizosphere, it was established that *Bacillus* sp. (13B41), *Advenella incenata* (22A67), *Pantoea dispersa* (22B45), and *Rhizobium pusense* (31B11) strains efficiently synthesized indoles, produced siderophores, and solubilized phosphates. Individual inoculation of these strains on corn plants showed a significant increase in several morphological parameters (height, dry weight) and chlorophyll content. For this reason, it is possible to use these bacteria for field experiments as well [54], but they are also effective in growing vegetables in greenhouses [55] and they also helped the phosphorus solubilization of *Lolium* and *Festuca* species (*Bacilus* sp. EhS7 strain) [56]. According to the measurements of Phares et al. [57], the amount of applied NPK fertilizer can also be reduced by certain strains from genera *Pseudomonas* in corn cultivation, and Ummara et al. [58] reported that the application of PGPB in corn also increases diesel oil tolerance. *Citrobacter freundii* strain LMG 3246, *Citrobacter braakii* DSM 17596, and strain G5 are compatible as PGPB and can be developed as biofertilizers [59]. Regarding living biotic systems, microbially produced organic ligands cannot noticeably affect the reactivity of magnesium–oxygen and calcium–oxygen sites on the diopside surface [60]. Rice, as another very important food industry crop, also shows good results in research projects based on PGPB utilization. Strains from genera *Bacillus*, *Pseudomonas*, *Enterobacter*, and *Streptomyces* greatly helped to improve and maintain the health of rice plants [61], but *Pseudomonas* sp. also plays a role in the development of Medicago sativa [62]. Metal-resistant PGPB increased canola biomass and total Cu uptake in canola stocks in Cu-contaminated agricultural areas near heavy metal mining tailings. The test bacterial strains *Burkholderia cepacia* J62, *Microbacterium oxydans* JYC17, and *Pseudomonas thivervalensis* Y1-3-9 had good growth-promoting ability and changed the bacterial consortium composition of the rhizosphere and endosphere of rapeseed. They increased the ascorbate–glutathione (AsA GSH) content of these plants and reduced the oxidative stress caused by heavy metals [63]. *Serratia* sp. AI001 and *Klebsiella* sp. AI002 strains had a beneficial effect on the uptake of cadmium by *Solanum tuberosum* [64]. PGPB can also be suitable for mitigating arsenic (As) pollution [65]. The reduction in the toxic effects of mercury has been confirmed for *Jeotgalicoccus huakuii* and *Bacillus amyloliquefaciens* bacteria [66]. These latest results have also been collected in tabular form (Table 1).

Bioprecipitation involves metals binding to the anionic functional groups of extracellular substances present on the surface of cells. Examples include sulfhydryl, amine, sulfonate, hydroxyl, carboxyl, and amide groups [67].

Prospectively, soil salinity will become a more and more serious issue in the coming years and will damage agricultural production, as it drastically affects microbial activity [43]. PGPB has various mechanisms to improve plant survival under saline conditions [68], such as increased sugar production, pigment number, and ascorbic acid [5]. Research knowledge about the molecular mechanisms of salt-tolerant PGPB to alleviate salt stress is still incomplete, but many studies are currently being conducted to learn about them. Salt-tolerant and halophilic PGPB can be a sustainable solution to improve the productivity of saline soils and play role in achieving the objectives related to food security and reducing the desertification of agro-ecosystems [68]. Rhizobacteria containing 1-aminocyclopropane-1-carboxylic acid deaminase (ACC deaminase) activity play a role in reducing plant stress tolerance [69]. ACC deaminase, which is produced by natural or genetically engineered PGPR in coexistence with different plants, can be considered as a significant environmental remediation approach for the foreseeable future to balance the ethylene level of plants under various abiotic stresses, such as abiotic stress or salt stress caused by climate change [70]. This enzyme is a direct precursor of ethylene biosynthesis [71]. ACC deaminase produces α-ketobutyrate and ammonia compounds. By reducing the level of ACC, these compounds prevent the high rate of synthesis of ethylene under stress conditions. This is one of the most effective mechanisms for inducing plant salt stress [72]. It can be said that many results have been achieved recently. ACC deaminase produced by *Bacillus subtilis* Rhizo SF 48 increased drought stress in tomato [73]. ACC deaminase produced by *Bacillus cereus* was also effective for tomatoes [74]. In case of drought, Enterobacter cloacae can also be used in corn cultivation with regard to ACC deaminase [75].

In addition to the increase in salinity, desertification is a significant problem. Zheng et al. [76] found that the species diversity of shrubs and herbs in rocky, desertified areas was significantly influenced by the microbiological composition of the soil. Among the bacteria, *Verrucomicrobium* sp. was significantly correlated with dry soil characteristics. MetaCyc analyses revealed that metabolic bacteria have the highest relative abundance in arid areas.

More and more publications are about which materials are also suitable for promoting the use of PGPB. Coumarins are one of the groups of plant-derived secondary metabolites formed via the phenylpropanoid pathway. They have an iron mobilizing effect, which is produced and secreted by the root system. Research shows the ever-widening results of coumarins in plant protection and in their role of promoting iron absorption [77]. The use of exogenous selenium is also very important in selenium-deficient areas, in order to improve stress tolerance. Selenium will continue to play a prominent role in the understanding of plant–soil interactions in the future [78]. Melatonin behaves particularly well in promoting plant PGPB relationships against biotic and abiotic stressors, increasing stress tolerance. During harvesting, the half-life of melatonin is an important factor—among other things in residual water treatments [79]. The use of microorganisms promotes a more sustainable and environmentally friendly approach to plant management practices [80]. Research indicates that PGPB can begin to replace other phytoremediation practices, such as inorganic fertilizers, phytohormones, and chelating agents, and corresponding costs will decrease as a result [81].

## 2. Conclusions

In summary, it can be said that the soil–microbiome relationship is an important and current field of science in our time. The widespread use of PGPB can be a solution for increasing and maintaining sustainable agricultural production with less environmental impact. The recent development of genetic technology also plays a role in increasing the potential of microorganisms and may accelerate their use in the phytoremediation of metal-contaminated soil. While significant advances have been made in transgenics in recent decades, the emergence of CRISPR technology will contribute even more to development and the creation of new research results. The simplicity and efficiency of this gene-editing technique can result in species-specific PGPB with enhanced ability to synthesize key compounds in the context of microbial-assisted phytoremediation. By using PGPB, a part of the use of artificial fertilizers, pesticides, and herbicides could be replaced; therefore, we could also contribute to the reduction in agricultural pollutions. As in another perspective of relevant studies, it can be stated that more and more bacterial strains are becoming suitable for use as PGPB. The projections described in the publications reveal that many new results may be obtained on the subject in the near future. Many soil–plant relationships are suitable for filtering out and breaking down polluting compounds that have entered the soil. Consequently, the research subject is important and continuous, and by applying and researching the PGPB, we can create a way to build a more sustainable future.

## Figures and Tables

**Table 1:** Recent research results on strains and species suitable as PGPB (Horotán, 2023).

Plant Species	Effects	PGPB	Ref.
*Arabidopsis thaliana* (L.) Heynh.	Affected root nutrient delivery system. Induce the genes related to nitrate and ammonium uptake and transfer.	*Bacillus* spp.	[45]
*Brassica napus* L.	Increased biomass and total Cu uptake. Growth-promoting. Reduce oxidative stress by heavy metals.	JYC17, Y1-3-9, J62	[63]
*Cucumis sativus* L.	Growth-promoting.	*Aspergillus niger* MJ1, *Pseudomonas stutzeri* DSM4166, *P. fluorescens* CHA0-nif	[46]
*Cucumis sativus* L.	Support for phosphorus supply.	*Enterobacter* 15S	[53]
*Festuca* spp. L.	Phosphorus solubilization.	*Bacilus* sp. EhS7 strain	[56]
*Impatiens walleriana* Hook.f.	Stimulated production of fatty acids, proteins, and amino acids. Promoted the formation of a higher proportion of oxidative proteins.	*Bacillus pumilus* W8, *B. pumilus* LZP02, *B. pumilus* JPVS11, *B. pumilus* TUAT-1, *B. pumilus* TRS-3, *B. pumilus* EU927414	[44]
*Lactuca sativa* L.	Growth-promoting.	*Aspergillus niger* MJ1, *Pseudomonas stutzeri* DSM4166 és *P. fluorescens* CHA0-nif	[46]
*Lolium* spp. L.	Phosphorus solubilization.	*Bacilus* sp. EhS7 strain	[56]
*Medicago sativa* L.	Growth-promoting.	*Pseudomonas* sp.	[62]
*Oryza sativa* L.	Improved and maintained health.	*Bacillus*, *Pseudomonas*, *Enterobacter*, *Streptomyces*	[61]
*Petunia x hybrida* (Sweet) D. Don ex W. H. Baxter	Stimulated production of fatty acids, proteins, and amino acids. Promoted the formation of higher proportion of oxidative proteins.	*Bacillus pumilus* W8, *B. pumilus* LZP02, *B. pumilus* JPVS11, *B. pumilus* TUAT-1, *B. pumilus* TRS-3, *B. pumilus* EU927414	[44]
*Solanum tuberosum* L.	Effect on cadmium uptake.	*Serratia* sp. AI001, *Klebsiella* sp. AI002 strain	[64]
*Spinacia oleracea* L.	Increased dry weight of the shoot. Strengthened vitality.	*Lysinibacillus* GIC41	[52]
*Viola x wittrockiana* Gams ex Nauenb. and Buttler	Stimulated production of fatty acids, proteins, and amino acids. Promoted the formation of higher proportion of oxidative proteins.	*Bacillus pumilus* W8, *B. pumilus* LZP02, *B. pumilus* JPVS11, *B. pumilus* TUAT-1, *B. pumilus* TRS-3, *B. pumilus* EU927414	[44]
*Zea mays* L.	Support for phosphorus supply.	*Enterobacter* 15S	[53]
	Yield increase. Higher protein and fiber content in the crop.	*Bacillus mojavensis, Bacillus subtilis, Bacillus pumilus, Bacillus pseudomycoides*	[19]
	Increased height, dry shoot, and root mass, and SPAD values.	*Bacillus* sp. (13B41), *Advenella incenata* (22A67), *Pantoea dispersa* (22B45), *Rhizobium pusense* (31B11)	[54]
	NPK fertilizer usage reduced. Increased diesel oil resistance.	*Pseudomonas* spp.	[57,58]

## Data Availability

No new data were created.
